# A Deep Learning Model for the Normalization of Institution Names by Multisource Literature Feature Fusion: Algorithm Development Study

**DOI:** 10.2196/47434

**Published:** 2023-08-18

**Authors:** Yifei Chen, Xiaoying Li, Aihua Li, Yongjie Li, Xuemei Yang, Ziluo Lin, Shirui Yu, Xiaoli Tang

**Affiliations:** 1 Institute of Medical Information Chinese Academy of Medical Sciences Beijing China

**Keywords:** multisource literature, institution name normalization, deep learning, bidirectional encoder representations from transformers, BERT

## Abstract

**Background:**

The normalization of institution names is of great importance for literature retrieval, statistics of academic achievements, and evaluation of the competitiveness of research institutions. Differences in authors’ writing habits and spelling mistakes lead to various names of institutions, which affects the analysis of publication data. With the development of deep learning models and the increasing maturity of natural language processing methods, training a deep learning–based institution name normalization model can increase the accuracy of institution name normalization at the semantic level.

**Objective:**

This study aimed to train a deep learning–based model for institution name normalization based on the feature fusion of affiliation data from multisource literature, which would realize the normalization of institution name variants with the help of authority files and achieve a high specification accuracy after several rounds of training and optimization.

**Methods:**

In this study, an institution name normalization–oriented model was trained based on bidirectional encoder representations from transformers (BERT) and other deep learning models, including the institution classification model, institutional hierarchical relation extraction model, and institution matching and merging model. The model was then trained to automatically learn institutional features by pretraining and fine-tuning, and institution names were extracted from the affiliation data of 3 databases to complete the normalization process: Dimensions, Web of Science, and Scopus.

**Results:**

It was found that the trained model could achieve at least 3 functions. First, the model could identify the institution name that is consistent with the authority files and associate the name with the files through the unique institution ID. Second, it could identify the nonstandard institution name variants, such as singular forms, plural changes, and abbreviations, and update the authority files. Third, it could identify the unregistered institutions and add them to the authority files, so that when the institution appeared again, the model could identify and regard it as a registered institution. Moreover, the test results showed that the accuracy of the normalization model reached 93.79%, indicating the promising performance of the model for the normalization of institution names.

**Conclusions:**

The deep learning–based institution name normalization model trained in this study exhibited high accuracy. Therefore, it could be widely applied in the evaluation of the competitiveness of research institutions, analysis of research fields of institutions, and construction of interinstitutional cooperation networks, among others, showing high application value.

## Introduction

### Background

The institution name is essential for describing the scientific research entity in scientific and technical literature. It is not only a key entrance for literature retrieval but also a vital statistical unit for the statistics of academic achievements and evaluation of the influence and competitiveness of scientific research institutions. However, differences in writing habits, spelling mistakes, space variations, use of abbreviations, etc, result in various name variants of the same institution in different publications, which may influence the analysis results of the publication data. It has been shown that Iranian universities fall behind in global rankings just because the authors used the nonstandard names of universities [1]. Consequently, the normalization of institution names has become a key issue in academic research.

The key to the normalization of institution names is the calculation of the similarity between different institution names. In most early studies, people often determined whether 2 different names refer to the same institution based on the literal similarity by 2 commonly used methods, namely, Edit Distance [2] and Jaccard Index [3]. Such methods may group 2 institutions with similar names together because of the high literal similarity. In recent studies, scholars have focused on the measurement of the semantic similarity of institution names and the recognition of institution name variants using machine learning and deep learning, which greatly improve the accuracy of institution name normalization [4].

In this study, a model for institution name normalization was trained by the fusion of multisource literature features based on deep learning to achieve the normalized management of institution names. We developed 4 submodels: an institution classification model, institutional hierarchical relation extraction model, zip code extraction model, and institution matching and merging model. On the basis of the results of the model normalization process, rules were established to enhance the model’s performance. Consequently, the model is capable of processing various types of affiliation data and can be effectively used in multiple domains. The performance of our model was found to be highly promising, with an overall accuracy rate of 93.79%, a recall rate of 93.08%, and an *F*_1_-score of 93.43%.

The focuses of this study are three-fold:

1. To correctly identify the institution name that matches the authority files and associate the name with the files by assigning the appropriate unique institution ID
2. To correctly recognize the name variants of the registered institutions, such as single and plural forms, the text transform of words, and the abbreviation of the institution names, and normalize them to canonical names
3. To correctly identify an unregistered institution and add relevant information to the authority files. When the institution appears again, the model treats it as a registered institution

This study innovates in the following aspects:

1. The model for institution name normalization was developed based on bidirectional encoder representations from transformers (BERT) and other deep learning models, which could be used to analyze and process the institutional data from multiple sources (Dimensions, Scopus, and Web of Science [WoS]) and construct authority files to normalize the institution name at the semantic level.
2. In this study, the entire process of institution name normalization was sorted out in detail, and the model for institution name normalization was designed, which includes submodels such as the classification model, hierarchical relation extraction model, and matching and merging model. These models work together to address the matching problem caused by the differences in the text transform and punctuation in the institution name and realize the specification of institution names in the complete sense. Thus, this method is worthy of widespread application and promotion.
3. Most of the existing studies on institution name normalization focus on coarse-grained normalization, that is, only the top-level institution names are regulated. This study achieved a fine-grained level of institution name normalization by extracting institution hierarchical relationships, which would regulate the institution names at each level, and the relationships between superior and subordinate institutions, making literature retrieval and statistics more convenient and faster.

### Related Works

#### Development of Institution Name Normalization

Numerous scholars have conducted research on institution name normalization and achieved promising performance. According to the key techniques for institution name normalization, these methods can be divided into 4 groups: the string similarity matching–based method, rule-based method, machine learning–based method, and deep learning–based method.

The string similarity matching–based method, dominated by the Edit Distance and Jaccard Index, has been applied to determine whether 2 names represent the same institution by comparing their literal similarity, thereby realizing the identification of 1 institution despite its different names. Edit Distance has been used to calculate differences in the strings of institution names and to recognize spelling mistakes and other name variants since 1997 [2]. Subsequently, James et al [3] further improved the clustering algorithm by combining the Jaccard Index and Edit Distance and clustered similar strings by replacing each string in the cluster with a standard form to achieve name normalization. Jacob et al [5] designed the sCooL system, which can be used to normalize institution names in the resumes using Levenshtein, N-gram, Jaccard similarity, and other algorithms. However, some issues cannot be addressed by relying solely on the string similarity matching–based method. For example, similar names of different institutions may easily cause mismatching.

The principle of the rule-based method is to manually build the rules using the author’s address information, institution attributes, and other features to recognize possible matching institution names based on the rules and eliminate the wrong matching pairs according to the threshold. To eliminate the limitations of string similarity matching, Huang et al [6] built a rule-based institution name disambiguation algorithm using data mining technology, which has reached a high precision in mathematics, psychology, and other fields. Huang et al [7] also established institution name matching rules by introducing the unique identifier of the institution and determined whether 2 names represent the same institution by comparing the Global Research Identifier Database ID, International Standard Name ID, and other identifiers. Some institutions have also conducted corresponding research. For example, the bibliometric database built by the Swedish Research Council based on WoS according to the rules was subjected to affiliation disambiguation for the matching of institutions, cities, addresses, etc. These rules can be used to process more than 99% of the Swedish address strings [8]. Generally, this type of method has poor portability owing to the manually developed rules.

Various machine learning models have been trained for institution name normalization and name disambiguation, such as naive Bayes, support vector machine (SVM), and K-means clustering. Han et al [9] compared the disambiguation effects of naive Bayes and SVM on the ambiguity caused by name abbreviations and spelling mistakes in different scenarios. Balsmeier et al [10] disambiguated the inventor’s name in the patent database using the K-means clustering algorithm.

Compared with the machine learning–based methods involving much human labor, the deep learning–based models may automatically learn the institutional features through less annotated data. He et al [11] established a deep neural network–based entity disambiguation model, which was used to measure the entity similarity in combination with the context and optimize the representations of entities and documents, thus achieving promising disambiguation results. With regard to the neglect of the word order in text by the Bag of Word model, Phan et al [4] firstly used long short-term memory (LSTM) and attention mechanism for entity disambiguation. Jiang et al [12] developed a Dual-Channel Hybrid Network model by fusing a convolutional neural network (CNN) model and a capsule model with a self-attention mechanism, outperforming the mainstream deep learning model in Chinese short text disambiguation. Entity normalization and disambiguation based on deep learning models have higher accuracy and recall rates than traditional methods and therefore deserve more research attention.

#### Deep Learning Models

On the basis of the initial success of machine learning, deep learning models have been widely used in speech recognition, image classification and search, natural language processing (NLP), and other fields. Deep learning models can learn features from the training data and exhibit a stronger data processing ability [13]. The institution name normalization mentioned in this study actually refers to the completion of NLP tasks using deep learning models. Common models mainly include CNN, recurrent neural network (RNN), LSTM, and BERT models. Previous developmental studies have demonstrated that both CNN and RNN models have showed their respective advantages in emotion analysis, entity recognition, part-of-speech tagging, and other NLP tasks [14]. Initially, the CNN model was primarily used for image processing and then applied to effectively mine the semantic information of the context [15], which is a typical NLP task. With a relatively simple internal architecture and low requirements for computing resources, the RNN model cannot effectively deal with the correlation between long sequences and thus easily causes a vanishing gradient or exploding gradient [16]. Therefore, the gating mechanism of LSTM was proposed to address the gradient issues.

In this study, we trained the normalization model of institution names developed on BERT, which is a pretrained language model that has been broadly used for entity extraction, text classification, emotion analysis, and other NLP tasks with the best performance [17]. With the architecture of a bidirectional transformer [18], BERT can complete parallel computing and thus greatly improve the operation efficiency. In addition, the core mechanism of the transformer, the self-attention mechanism, enables the BERT model to pay more attention to the valuable information among the input data and assign different weights to the words by fully learning contextual features [19]. The BERT model has been broadly applied to named entity recognition (NER) tasks and provides promising results [20,21]. Commonly used pretrained language models include generative pretrained transformer, Global Vectors for Word Representation (GloVe), and Embeddings from Language Models. As the BERT model is state-of-the-art in many NLP tasks [22] and the study [23] shows that the BERT-based model is easily fine-tuned and can be applied to any form of entity name normalization in the biomedical field, it was adopted in this study. It could empower this study and enable the institution name normalization model to achieve higher accuracy.

#### NER Tasks

NER is a common NLP task. Initially, NER was mainly used for string matching based on dictionaries and manual rules, but it showed poor feasibility and portability when dealing with complex data [24]. With the deepening of research, machine learning–based methods have been gradually adopted in NER, such as Hidden Markov Model [25], conditional random field (CRF) [26], and SVM [27] models, but they require much manual work. Deep learning methods are widely used in NER tasks. Hammerton et al [28] first used LSTM to complete NER tasks with data from the Reuters Corpus and the European Corpus Initiative Multilingual Corpus, and Peng et al [29] proposed the LSTM-CRF model on the basis of improved existing models and methods, which has significantly optimized the word segmentation effect compared with traditional methods. Lample et al [30] proposed a neural network model combining bidirectional LSTM (BiLSTM) and CRF, and the context sequence information can be obtained with this bidirectional structure that has been widely used in NER tasks. To further recognize the overlapping nested entities in sentences, span-based methods have been proposed in the NER field. For example, Mandar et al [31] proposed a pretrained model, SpanBERT, which was experimentally demonstrated to generally outperform the BERT model in terms of relation extraction and coreference resolution. The extraction of institutional hierarchical relationships is based on the accurate identification of institution names. In this study, we used the NER model to construct the institutional hierarchical relation extraction model.

## Methods

### Ethical Considerations

Ethics approval was not required because the data processed in this study are publicly available literature data, including article titles, institutional addresses, institution names, etc, with no user privacy involved.

### Study Overview

**Figure 1:**
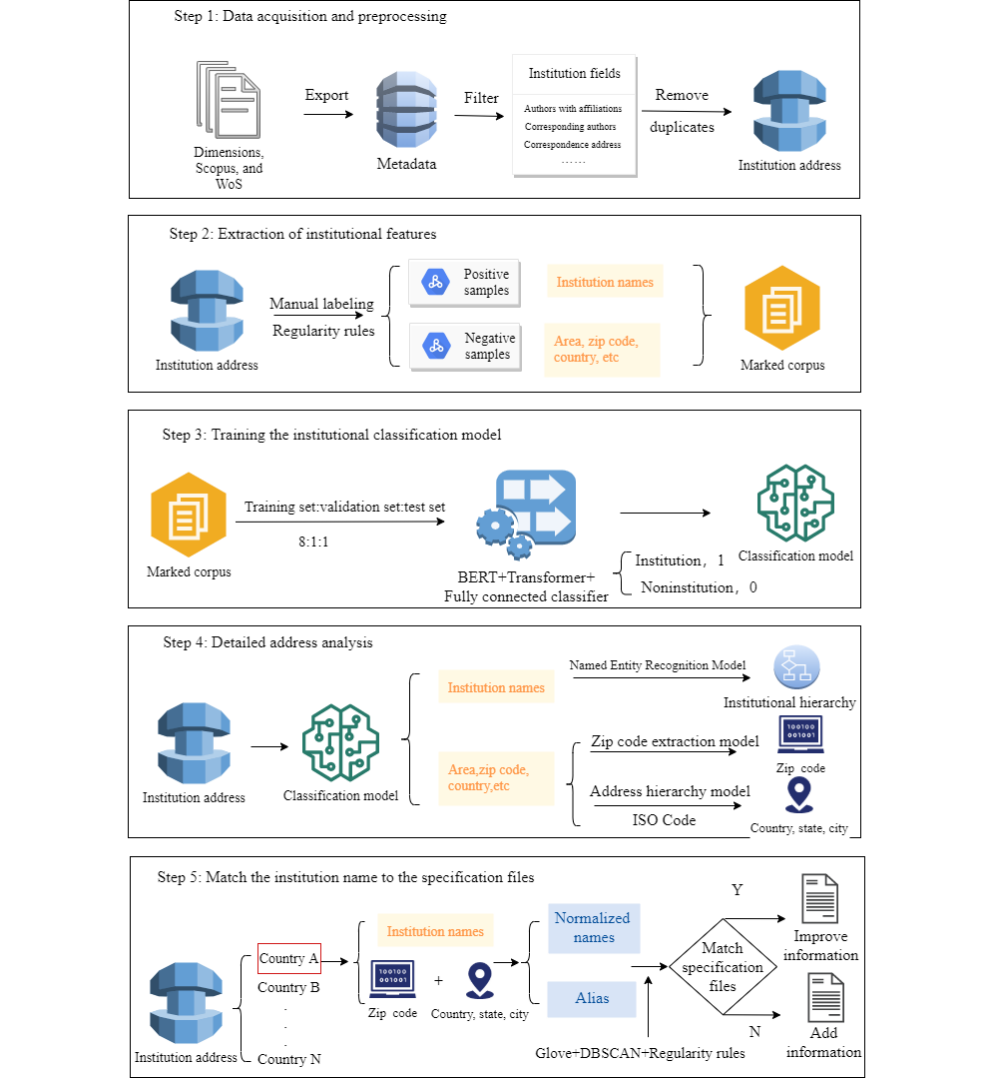
Research route. BERT: bidirectional encoder representations from transformers; DBSCAN: Density-Based Spatial Clustering of Applications with Noise; GloVe: Global Vectors for Word Representation; ISO: International Organization for Standardization.

To improve the effectiveness of institutional name normalization, we proposed a 4-stage institutional name normalization model applicable to multiple sources and types of literature data. In this study, the entire process involves 4 models: the institution classification model, institutional hierarchical relation extraction model, institution zip code extraction model, and institution matching and merging model. The research route is shown in Figure 1, and the process is as follows: first, the affiliation data were obtained from multisource literature databases; second, information about the institution, including the name, address, and hierarchical relation, was extracted from the affiliation data using the BERT model; and third, the institution data were clustered and merged through the clustering algorithm and then compared with the normalized institution names in authority files. This process not only helped realize the normalization of different names and unify the identification of the same institution but also contributed to the update and improvement of the authority files of the institution.

### Analysis of Multisource Literature Features

The model for institution name normalization developed on deep learning in this study could be used to simultaneously process the affiliation data from the Dimensions, WoS, and Scopus databases, greatly enhancing its practical application value in institution name normalization. Specifically, the literature data were collected from Dimensions, WoS, and Scopus (including 49 fields) at the same time as per the literature collection requirements, and data from different sources had different field types (the fields required by institution name normalization are indicated in italics in Table 1). In addition, data filtering was performed to eliminate duplicate and invalid data, and the fields required by institution name normalization were filtered for cleaning again to obtain the initial data set of the institution name normalization.

Difficulties in multisource data processing were mainly reflected in two aspects: (1) the types and quantities of data fields from different sources were nonuniform, which increased the difficulty of analysis, and (2) we randomly selected a field. Even if the field types of data were the same, the presentation forms of the different databases were quite different. Taking the article “Model of Ischemic Heart Disease and Video-Based Comparison of Cardiomyocyte Contraction Using hiPSC-Derived Cardiomyocytes” as an example, we randomly selected the authors’ affiliations as the comparison field. The content of the field from Dimensions is represented as “Yun, Liu(Okayama University); Yin, Liang(Okayama University); Mengxue, Wang(Okayama University); Chen, Wang(Okayama University); Heng, Wei(Kyoto University); Keij, Naruse(Okayama University); Ken, Takahashi(Okayama University),” whereas the content of the same field from WoS is represented as “[Liu, Yun; Liang, Yin; Wang, Mengxue; Wang, Chen; Naruse, Keiji; Takahashi, Ken] Okayama Univ, Dept Cardiovasc Physiol, Grad Sch Med Dent & Pharmaceut Sci, Okayama, Japan; [Heng, Wei] Kyoto Univ, Inst Lab Anim, Grad Sch Med, Kyoto, Japan,” and the content from Scopus is represented as “Liu, Y., Department of Cardiovascular Physiology, Graduate School of Medicine, Dentistry and Pharmaceutical Sciences, Okayama University, Japan; Liang, Y., Department of Cardiovascular Physiology, Graduate School of Medicine, Dentistry and Pharmaceutical Sciences, Okayama University, Japan; Wang, M., Department of Cardiovascular Physiology, Graduate School of Medicine, Dentistry and Pharmaceutical Sciences, Okayama University, Japan; Wang, C., Department of Cardiovascular Physiology, Graduate School of Medicine, Dentistry and Pharmaceutical Sciences, Okayama University, Japan; Wei, H., Institute of Laboratory Animals, Graduate School of Medicine, Kyoto University, Japan; Naruse, K., Department of Cardiovascular Physiology, Graduate School of Medicine, Dentistry and Pharmaceutical Sciences, Okayama University, Japan; Takahashi, K., Department of Cardiovascular Physiology, Graduate School of Medicine, Dentistry and Pharmaceutical Sciences, Okayama University, Japan.” Upon observing these 3 fields, it is evident that there are significant differences in their contents. If the reliability of data from the 3 databases was different, the priority should be Scopus, followed by Dimensions and WoS. When an article was included in multiple databases at the same time, the model further processed the article according to the priority ranking.

**Table 1:** Multisource literature fields.

Source	Fields
Dimensions	*WoS*^a^*DOI*^b^*, PMID*^c^*, PMCID*^d^*, Title, Abstract, Authors-D, Authors (Raw Affiliation), Corresponding Authors, Authors Affiliations, Research Organizations—standardized, GRID*^e^*IDs, City of Research organization, State of Research organization, Country of Research organization,* Acknowledgments, Source Title, Publisher, PubYear, MeSH^f^ terms, Publication Date (web), Publication Date (print), Publication Type, Funder, Funder Group, Funder Country, UIDs^g^ of Supporting Grants, Supporting Grants, and Times Cited
Scopus	*Authors-S (Authors–Abbreviated Source), Authors with affiliations, Correspondence Address, Affiliations, Authors ID,* Funding Text 1, and Document Type
WoS	*AF*^h^*, AU*^i^*, C1*^j^*, RP*^k^*,* RI^l^, OI^m^, DT^n^, LA^o^, FU^p^, PI^q^, PA^r^, WC^s^, SC^t^, and UT^u^

^a^WoS: Web of Science; the fields required by institution name normalization are indicated in italics.

^b^DOI: digital object unique identifier.

^c^PMID: PubMed unique identifier.

^d^PMCID: PubMed central identifier.

^e^GRID: Global Research Identifier Database.

^f^MeSH: Medical Subject Headings.

^g^UIDs: unique identifiers.

^h^AF: author full name.

^i^AU: author.

^j^C1: author address.

^k^RP: reprint address.

^l^RI: Researcher ID.

^m^OI: ORCID identifier.

^n^DT: document type.

^o^LA: language.

^p^FU: funding agency and grant number.

^q^PI: publisher city.

^r^PA: publisher address.

^s^WC: Web of Science categories.

^t^SC: research areas.

^u^UT: accession number.

### Deep Learning Model for Institution Name Normalization

**Figure 2:**
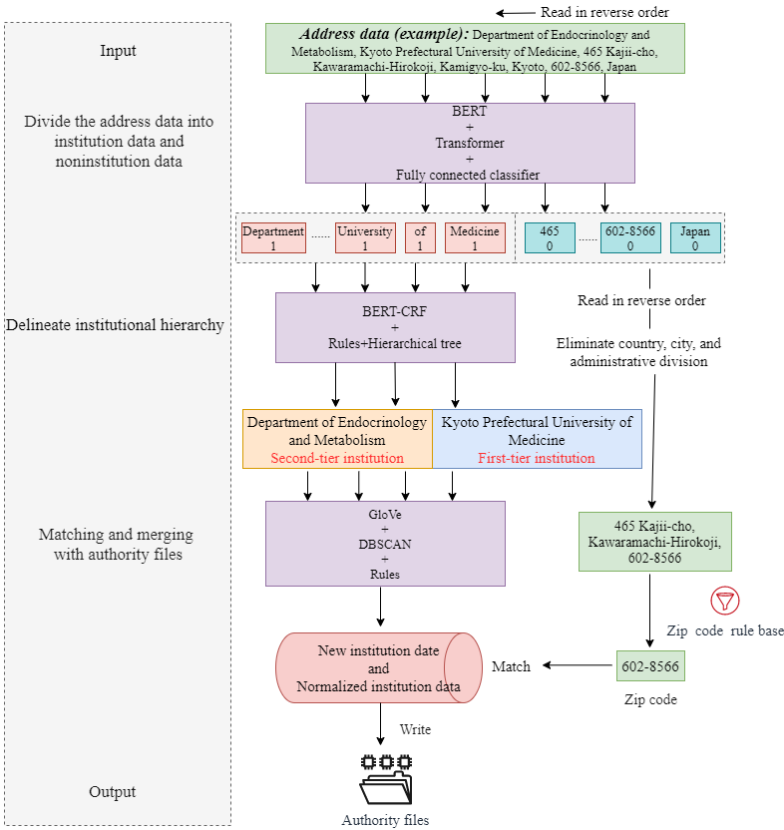
Models’ architecture. BERT: bidirectional encoder representations from transformers; CRF: conditional random fields; DBSCAN: Density-Based Spatial Clustering of Applications with Noise; GloVe: Global Vectors for Word Representation.

The complete architecture of the institution name normalization model is shown in Figure 2 and is explained in detail in the following 4 parts of this section.

#### Institution Classification Model

The affiliation data from the literature database contain a large number of institution names and attribute information, and it is difficult for the machine to distinguish institutional information from noninstitutional information. Therefore, the accurate extraction of institutional names is the first step in institution name normalization. Owing to the complex and variable forms of addresses extracted from the affiliation data, this field often contains multitier institutions and detailed addresses of the institutions (including the information on street, zip code, region, and country). For example, in “Division of Biology and Biological Engineering, California Institute of Technology, 1200 E. California Blvd., MC 114-96, Pasadena, CA 91125, USA,” it is difficult to recognize the institution name accurately according to the general rules. Considering that the specific keywords and syntactic structures of institution names differ from the detailed address information, a supervised learning–based classifier model was developed in this study to recognize the address information as institutional phrases and noninstitutional phrases through automatic learning of institutional features, so as to extract institution names.

The accurate separation of institutional and detailed address information (noninstitutional part) from the original address is essentially a binary classification problem, specifically for short texts. In the process of institution name recognition, a machine’s understanding of word semantics is crucial. The institution classification model consists of a pretrained BERT model, transformer, and fully connected classifier. Considering that the institution name usually appears before the noninstitutional information (detailed address) in the full address field, the model uses a reverse classification method to segment the original address. That is, it identifies each word in reverse order until it reads a word that represents institutional information and then stops classifying. At this point, the first half of the address is considered to be institutional information and the second half is considered to be noninstitutional information. The BERT [32] model is a deep learning–based language representation model and can provide richer semantic information of words (especially keywords), such as “Institute,” “Department,” and “University,” by virtue of its multilayer transformer and the ability of converting the input texts into word vector representation. The transformer encodes the word order and background information of a word in a phrase. For example, the institutional hierarchical order and the differences between institutional and noninstitutional features can be encoded by the transformer. At present, the commonly used encoders include transformer [18], BiLSTM [30], and CNN [33]. The transformer incorporates a self-attention mechanism that focuses on the valuable information among the input data, which are beneficial for encoding important words; thus, it was adopted as an encoder herein. The output result from the transformer is mapped into a dichotomy by a fully connected classifier, with 0 representing noninstitution and 1 representing institution, thereby achieving the separation of institution names and detailed addresses.

The training process of the model is as follows:

1. According to the regular rules, the institution affiliation data collected and cleaned will be divided into positive samples (institutional phrases) and negative samples (noninstitutional phrases such as detailed address, region, and zip code) and used as the manual annotation corpus required for the training. The data size of corpus is 33,379, with 22,927 positive samples and 10,452 negative samples.
2. The annotated corpus was divided into training, validation, and testing sets in a ratio of 8:1:1. Then, the training set was entered into the model to enable the model to automatically learn institutional features, by which a trained institution classification model could be obtained. After many rounds of training and optimization, the precision of the model can reach >94%, indicating that it can be used to accurately distinguish the institution name and address.

After training, when a phrase was entered, such as “California Institute of Technology,” the model returned “True” (institution) or “False” (noninstitution), and when an original address was entered, the model split it into detailed address and institution information. The operating effects of the institutional classification model are shown in Table 2.

**Table 2:** Model classification results.

Original address field	Address information	Institution information
Institute of Hematology, General Medical Center, Blood Diseases Hospital, Chinese Academy of Medical Sciences and Peking Union Medical College, Tianjin, China	Tianjin, China	Institute of Hematology, General Medical Center, Blood Diseases Hospital, Chinese Academy of Medical Sciences and Peking Union Medical College
Medical Research Council Population Health Research Unit, Nuffield Department of Population Health, University of Oxford, Oxford, United Kingdom	Oxford, United Kingdom	Medical Research Council Population Health Research Unit, Nuffield Department of Population Health, University of Oxford
Department of Epidemiology, Peking University Health Science Center, Beijing, China	Beijing, China	Department of Epidemiology, Peking University Health Science Center
Institute of Population Health Sciences, Queen Mary University of London, London, United Kingdom	London, United Kingdom	Institute of Population Health Sciences, Queen Mary University of London
Department of Prosthodontics, Peking University School and Hospital of Stomatology and National Clinical Research Center for Oral Diseases and National Engineering Laboratory for Digital and Material Technology of Stomatology and Beijing Key Laboratory of Digital Stomatology, 22 Zhongguancun South Avenue, Haidian District, Beijing, 100081, China	22 Zhongguancun South Avenue, Haidian District, Beijing, 100081, China	Department of Prosthodontics, Peking University School and Hospital of Stomatology and National Clinical Research Center for Oral Diseases and National Engineering Laboratory for Digital and Material Technology of Stomatology and Beijing Key Laboratory of Digital Stomatology
Liuyang Center for Disease Control and Prevention, Liuyang, Hunan Province, China	Liuyang, Hunan Province, China	Liuyang Center for Disease Control and Prevention

#### Institutional Hierarchical Relation Extraction Model

Hierarchical relation is embedded in the institution information. For example, in “Medical Research Council Population Health Research Unit, Nuffield Department of Population Health, University of Oxford,” the “University of Oxford” is the first-tier institution, “Nuffield Department of Population Health” is the second, and “Medical Research Council Population Health Research Unit” is the third. In this study, an institutional hierarchical relation extraction model was constructed with a structure of the NER model plus rules plus hierarchical tree to maximize the effectiveness from multiple perspectives, such as rules and semantics, and normalize institutions at a finer granularity. First, the NER model sequentially labels the original address field with the Beginning Inside and Outside method. Once the labeling is completed, the deep learning model identifies the distinguishing features of institutions across different levels and provides an output for the labeled address field. At this stage, the institution-related information in the original address is labeled based on the primary, secondary, and tertiary levels. To ensure the precise extraction of hierarchical structures, this study also incorporates rules and hierarchical trees, which will be further explained in the latter part of this section.

It is worth noting that most existing techniques for institution name normalization and entity disambiguation operate at a broad level, with fewer studies focusing on the subdivision of institutional hierarchies. In this study, the model not only normalizes the names of institutions at each level but also finely organizes the hierarchical relationships among primary, secondary, and tertiary institutions, which is convenient for researchers to use the hierarchical structure of institutions for literature retrieval and statistics.

**Figure 3:**
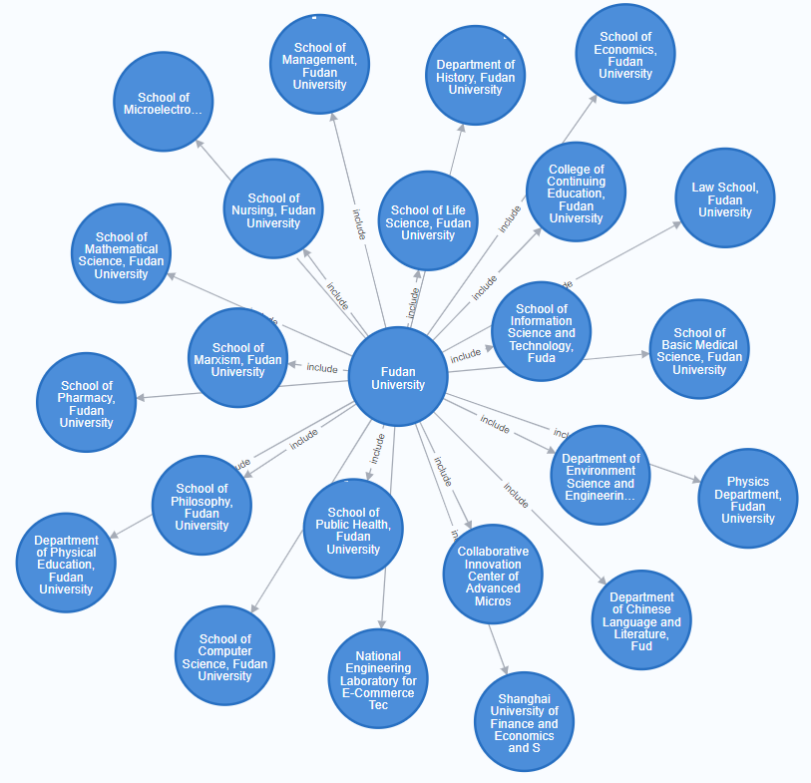
Hierarchical data construction using the Neo4j database.

The X-CRF framework was adopted as the main model for institution extraction, as this task does not involve the issue of span overlapping. The BiLSTM-CRF and BERT-CRF models were used in the specific experiments for the institutional NER. The experimental results showed that the effect of the BiLSTM model was restricted by the training data, resulting in inaccurate labels predicted by the model on the one hand and out of vocabulary when a word was used as a token on the other hand. Therefore, in this study, a large-scale pretrained BERT model with word pieces as its token was adopted to obtain text representation, which alleviated out of vocabulary problems to some extent. The institutional hierarchical relation in the address was extracted using the BERT-CRF model, and the institutional span and label were obtained. However, there might be span deviation, label error, institutional deficiencies and redundancies, or other problems in the extraction results. Therefore, in this study, the model’s prediction results were corrected primarily through the use of rules and hierarchical trees. The formulated rules include the institution name completion rule and the institutional order correction rule. The former determines whether the extracted content is a complete field, typically by checking whether it ends with a comma. For instance, the institution name is “University of Antwerp (Campus Drie Eiken),” but the NER model only extracts “University of Antwerp.” Meanwhile, the latter corrects the institutional hierarchical order in original addresses that are not standardized, such as “Queen Mary University of London, Institute of Population Health Sciences,” by adjusting the order according to the characteristic words of each level of institutions. Once the rules were manually established, they were incorporated into the model. Finally, for the special hierarchical relationships that cannot be identified by the machine, the institutional hierarchical relationships are imported into the model by constructing institutional hierarchical trees to complement the extraction results of the model. The data size in this study was small; therefore, the hierarchical tree was constructed using the open Neo4j database to correct the institutional hierarchical order. The constructed hierarchical relationship is shown in Figure 3.

#### Zip Code Extraction Model

The zip code extraction model was applied to extract the zip code from the address information and integrate it with other affiliation data. In the process of canonical information matching, the zip code information plays an auxiliary role in determination. As mentioned in the section of *Institution Classification Model* before, the institution classification model divides the original address into 2 parts: institutional information and noninstitutional information. The noninstitutional information may contain city, zip code, administrative division, and country. First, the model reads the noninstitutional information in reverse order with the help of the International Organization for Standardization (ISO) standard; eliminates the country, city, administrative division, etc; and then manually researches the coding rules of zip codes of different countries (eg, the zip code of China consists of 6 consecutive digits), builds a zip code rule base, and extracts the zip code from the remaining information, which has been tested to be more accurate than directly extracting the zip code. The specific process for extracting zip code is as follows:

1. High-quality websites, including those with more complete zip codes, were filtered and selected through manual searches.
2. The structure of the target website was analyzed, and zip code data were obtained using requests+concurrent futures, asynchronous programming, or other web crawler techniques. Then, the data obtained by the web crawler were cleaned, and invalid data were eliminated by lxml, BeautifulSoup, and regular expressions to obtain effective data for the construction of the zip code library.
3. As the zip code rules of each country are different, a country+zip code rule base was constructed manually to split the data after cleaning and to obtain the retrieval vector. Next, country information in the retrieval vector was obtained from the national database, zip code rules were obtained based on the country information, and zip codes were finally obtained according to the rules.

The extraction results are shown in Multimedia Appendix 1. The zip code rules for China, the United States, the United Kingdom, and Germany were included in the database; thus, the zip code extraction model achieved an accuracy of almost 100%.

#### Institution Matching and Merging Model

The institution name extracted from the institution address will be compared and matched with the name in authority files, which are computer-identifiable documents consisting of specification records. If the name matches the institution name in the authority files, then the institution will become a registered institution, and its specification information, such as the new alias, zip code, and official website, can be completed in authority files; otherwise, the institution is unregistered and will be included in the authority files as a new institution after manual review. In the process of matching and merging, the recall rate using traditional matching methods is relatively low because of the differences in text transform, punctuation, and word expression order among the institution names. In this study, the institution matching and merging model was developed using country grouping and vector clustering to improve the matching recall rate and reduce the running time of the model. The specific process is as follows: all registered and unregistered institutions are divided by countries, and rules are established for the preliminary processing of unregistered institutions, which include converting the names to lowercase for text vectorization, and eliminating nonalphabetic characters other than “&” in names and converting “&” to “and.” The GloVe model was used to transform the processed data into vectors, and the Density-Based Spatial Clustering of Applications with Noise (DBSCAN) algorithm was then used to cluster institution names under the same country. Subsequently, the new unregistered organizations are added or aliases of registered organizations are included in the authority files based on the clustering results. This operation can achieve multithreaded matching of different countries, greatly improving the matching efficiency.

Text representation is a critical step in the text clustering process, and the method and effect of text representation have a great influence on the model clustering effect. The most commonly used methods in text representation include bag of word and term frequency–inverse document frequency, which perform well in classification and clustering tasks; however, there are still some problems, such as an extremely high vector dimension, sparse data, failure to focus on the word order in sentences, and failure to learn text semantic information [34,35]. Multiple word embedding representation methods have been developed to overcome this limitation, such as Word2Vec [36], GloVe [37,38], and Embeddings from Language Models [39], which can effectively address the semantic problems of words in the text. In this study, GloVe was applied for text representation owing to its advantages of high accuracy and a short training period.

The classical clustering algorithms include K-means, Gaussian mixture model, and DBSCAN. For the K-means and Gaussian mixture models, the number of clusters must be set manually, and the same institution cannot be clustered automatically. However, the DBSCAN algorithm speeds up a rapid clustering and requires no manual setting for the number of clusters [40]. Moreover, the fault tolerance rate of the model can be reduced by setting a shorter intercluster distance when the DBSCAN algorithm is used. Therefore, the DBSCAN algorithm was applied in this study. The normalization of institution names was achieved using vector clustering. When an institution name vector cluster contains both registered and unregistered institutions, the unregistered institutions are grouped under the registered ones and listed as aliases in authority files. In cases where the cluster only contains unregistered institution names, the institution name with the largest number is taken as the base data for grouping and written in authority files after manual review.

In summary, the institution matching and merging model consists of 3 components: GloVe, DBSCAN, and a set of rules. First, we manually formulated rules to transform and process the data. Next, the organization names were represented as vectors using GloVe. Finally, the DBSCAN algorithm clusters the vectors, and the institution names are matched and merged with the authority files according to the clustering results. These 3 components work together to guarantee the precision of institution name matching while maintaining high operational efficiency.

## Results

### Qualitative Analysis

In this study, we combined the affiliation data from WoS, Dimensions, Scopus, and initially constructed institutional authority files through cleaning, clustering, and other normalization processes, which include not only English and Chinese normalized names, English and Chinese aliases but also the countries where institutions are located. To verify the accuracy of institution name normalization, the affiliation data of 129 articles were obtained from Dimensions, WoS, and Scopus, and a total of 665 institution names were extracted from them. Of the 129 articles, 100 were the latest published literature in January 2023, and were excluded from the training data. They were cleaned, deweighted, and imported into the model for analysis, thus normalizing the institution names. We invited 3 librarians to review the data item by item; 2 of them performed a back-to-back review and then asked the third person to review when there was a discrepancy between the 2 review results, and the review results of the 3 people were unified. The model achieved excellent results, with a 93.79% accuracy rate, 93.08% recall rate, and 93.43% F_1_-score. Here is a success example: the affiliation data are “Plastic Surgery Hospital, Chinese Academy of Medical Sciences and Peking Union Medical College, Beijing,100144, China.” The trained model could exactly extract the institution name and normalize it to the “Plastic Surgery Institute and Hospital, Chinese Academy of Medical Science and Peking Union Medical College.”

The three main functions that could be achieved by the model are outlined in the *Background* section and analyzed in depth as follows:

1. Identification of registered institutions: Once the name of a given institution from publications matched the name in authority files, the model could not only accurately identify the institution but also associate it with the corresponding institution in authority files. In addition, a unique ID was assigned according to the authority files.
2. Identification of the aliases of registered institutions: Although the input institution names are actually the same institution but have different expression forms, the model can identify such irregularly written name variants, accurately associate them with the corresponding institutions in authority files, and add aliases and affiliations to them (eg, the correct form of the author’s institution is “Chinese Academy of Medical Sciences & Peking Union Medical College,” but it is often written as “Chinese Academy of Medical Science & Peking Union Medical College”). It is worth noting that many institutions have very similar names, such as “Chinese Academy of Medical Sciences” and “China Academy of Chinese Medical Sciences.” They have similar names but actually point to different institutions. Mismatches of institution names are prone to occur if clustering is based solely on literal similarity. The deep learning model built in this study helped recognize institution names at the semantic level, which could identify the name variants of the same institution and effectively avoid mismatches between different institutions.
3. Identification of unregistered institutions: If the model identifies the input institution as an unregistered institution, a new institution would be created in authority files, and the information of normalized names and institution attributes can be completed with the help of the institution’s official website and other web resources. When the same entity reappeared, regardless of its normalization name or alias, the model could associate it with the corresponding new entity in authority files rather than treating it as an unregistered entity.

### Error Analysis

Error analysis is critical for understanding the model shortcomings, thereby contributing to the in-depth analysis and improvement of the model [41]. The model of institution name normalization achieved satisfactory results; however, 46 errors remained. We analyzed the specification of 665 institution names and then observed the error types and causes of errors to better optimize the model. The error analysis results are shown in Table 3.

**Table 3:** Error analysis results.

Error type	Reason	Improvement direction
Ambiguous institution aliases in authority files	One of the aliases of this secondary institution in authority files is “School of Life Science,” which is the same as the aliases of several secondary institutions, resulting in a mismatch of models.	Improve the authority files and increase the discrimination of the secondary institution aliases to avoid the appearance of the same or similar aliases.
Hierarchical relationship of nested institutions were not identified	Nested institution is a special type of institutional entity. It is necessary to specifically train a model that recognizes the relationships of nested institutions in order to accurately identify them. However, this study doesn’t train such a model.	Train a model that can recognize hierarchical relationships of nested institutions.
Missing identification of institution names	Artificial matching rules are not perfect enough, which affects model matching.	Further refine the matching rules and prepare more corpus for model training.

As mentioned in *Deep Learning Models* section, the deep learning–based model performed well overall, but there were still some issues, mainly in the following three aspects (Table 3):

1. The aliases in the specification document are ambiguous, which may cause the model to be matched incorrectly.
2. Some of the hierarchical relationships among nested institutions were not correctly identified.
3. The model sometimes failed to identify all the institution names, especially when there were a considerable number of institutions co-occurring in 1 article.

In view of the abovementioned problems, the follow-up work will focus on 3 aspects: first, further improve the authority files and the model to avoid the occurrence of ambiguous aliases, and if the model cannot accurately identify the parent institution owing to the similarity of institution aliases, a hint should be given to guide the technical staff to further review; secondly, train an institution classification model for nested institutions to further expand the matching range of the model and improve its matching accuracy; and third, adjust the matching rules to cover all possible address types to the maximum extent so that the institution names at each level can be correctly and fully matched.

## Discussion

### Major Applications of the Proposed Model

The deep learning–based model for institution name normalization trained in this study could be widely applied for the evaluation of institutions’ scientific research competitiveness, analysis of institutions’ research fields, and creation of interinstitutional cooperation networks. Accurate and comprehensive publication data form the basis of econometric analysis, and both the evaluation of the competitiveness of research institutions and the analysis of research fields require scientific econometric data. The former usually requires statistics on various indicators, such as the number of articles published by institutions, the citation frequency of papers, and the number of paper awards, whereas the latter can analyze the development trend of institutions by counting the fields in which their articles are published and cited more frequently. The accuracy of data statistics is based on the normalization of institution names. The normalization can effectively reduce errors in institutional academic achievement statistics and improve the quality and credibility of evaluation and analysis.

Interinstitutional scientific cooperation is an integral part of promoting scientific communication, which will help researchers discover cooperative relationships and spatial distribution and further analyze the core institutions in a research field using network indicators such as network density and betweenness centrality [42]. Commonly used tools include CiteSpace (Chaomei Chen), VOSviewer (Centre for Science and Technology Research, University of Leiden), and Pajek (Vladimir Batagelj and Andrej Mrvar). Generally, literature citation data are directly imported into the aforementioned software for the analysis and mapping of the cooperation network. However, owing to nonnormalized institution names, the resources of the same institution might be displayed as different institutions, which is not conducive to the integration and use of resources. Normalizing the names of institutions effectively avoids this issue and makes the interinstitutional cooperation relationship clearer and more explicit.

### Limitation and Future Work

A potential limitation of this study is that the extraction of hierarchical relationships for nested entities falls short of expectations. The structure of nested entities exhibits a unique pattern in which multiple entities are intertwined and nested within their names. As entity recognition usually involves assigning starting and ending markers to the entities, the intertwining of entities makes the marking process extremely challenging. The identification of nested entities presents a challenge for current NER tasks. In the near future, we will further optimize the model in terms of the classification of nested institutions’ affiliation data, try to construct a model for nested institution hierarchical relationship extraction, and then count the academic results of research institutions to actually validate the model.

### Conclusions

A unified and accurate description of institutional names is of great significance for the precise attribution of scientific research results, evaluation of the competitiveness of scientific research institutions, and even knowledge discovery. In this study, a deep learning–based model for institution name normalization was trained based on the integration of several submodels, such as the classification model, matching model, and merging model. The proposed model could accurately extract institution names and other information from multisource affiliation data by matching with the authority files and realize the normalization of institution names. After several rounds of testing, we found that the model could achieve 93.79% accuracy and has a promising specification effect, which would be widely used in downstream tasks such as institutional research field analysis and institutional influence assessment.

## Appendix

Multimedia Appendix 1 Zip code extraction results.

## Abbreviations

BERT: bidirectional encoder representations from transformers
BiLSTM: bidirectional long short-term memory
CNN: convolutional neural network
CRF: conditional random field
DBSCAN: Density-Based Spatial Clustering of Applications with Noise
GloVe: Global Vectors for Word Representation
ISO: International Organization for Standardization
LSTM: long short-term memory
NER: named entity recognition
NLP: natural language processing
RNN: recurrent neural network
SVM: support vector machine
WoS: Web of Science

## Data Availability

The data can be made available upon reasonable request, and please contact the corresponding author for further details. The authors would like to assure the readers that all data have been secured in accordance with the policies of the research institution and that the results have been reported accurately.
