# Supplementary figures and images for: A Deep Learning Model for the Normalization of Institution Names by Multisource Literature Feature Fusion: Algorithm Development Study
Source: JMIR Form Res. 2023 Aug 18;7:e47434. doi: 10.2196/47434 (PMC10474509; doi:10.2196/47434)

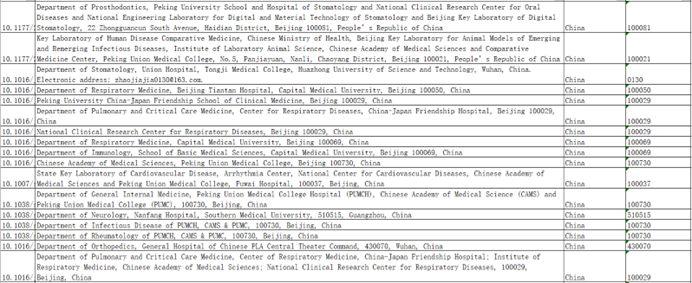

Supplement: Multimedia Appendix 1 [file formative_v7i1e47434_app1.png]
